# Establishing and Applying a Schistosomiasis Early Warning Index (SEWI) in the Lower Yangtze River Region of Jiangsu Province, China

**DOI:** 10.1371/journal.pone.0094012

**Published:** 2014-04-04

**Authors:** Kun Yang, Jun-Fang Xu, Jian-Feng Zhang, Wei Li, Jian He, Song Liang, Robert Bergquist

**Affiliations:** 1 Jiangsu Institute of Parasitic Diseases, Key Laboratory of Parasitic Disease Control and Prevention (Ministry of Health), Jiangsu Provincial Key Laboratory of Parasite Molecular Biology, Wuxi, Jiangsu Province, China; 2 Medicine school, Hubei University for Nationalities, Enshi, Hubei Province, China; 3 Department of Environmental and Global Health, College of Public Health and Health Professions, University of Florida, Gainesville, Florida, United States of America; 4 Emerging Pathogens Institute, University of Florida, Gainesville, Florida, United States of America; 5 Ingerod 407, Brastad, Sweden; University of Minnesota, United States of America

## Abstract

**Background:**

China has made remarkable progress in schistosomiasis control over the past decades. Transmission control has replaced morbidity control as the country moves towards the goal of elimination and the current challenge is to find a sensitive measure capable of gauging transmission risk in low-prevalence areas. The study aims to develop a Schistosomiasis Early Warning Index (SEWI) and demonstrate its use in Jiangsu Province along the lower Yangtze River.

**Methodology/Principal Findings:**

The Delphi approach, a structured communication technique, was used to develop the SEWI. Two rounds of interviews with 30 public health experts specialized in schistosomiasis control were conducted using 40 indicators that reflected different aspects of schistosomiasis transmission and control. The necessity, feasibility, and sensitivity of each indicator were assessed and the weight value of each indicator determined based on these experts' judgment. The system included 3 first-order indicators, 7 second-order indicators, and 30 third-order indicators. The 3 first-order indicators were *endemic status*, *control measures*, *social and environmental factors*, with the weight values 0.366, 0.343 and 0.291, respectively. For the 7 second-order indicators, the highest weight value was for *control measures for snails* (0.175) and the lowest for *transmission route* (0.110). We estimated and mapped the SEWI for endemic areas at the county scale in Jiangsu Province finding that the majority of the endemic areas were characterized as medium transmission risk (SEWI risk values between 0.3 and 0.6), while areas where transmission interruption had been officially declared showed SEWI values <0.30. A few isolated areas (e.g. endemic islands in the Yangtze River) produced SEWI values >0.60. These estimates are largely in agreement with the endemicity levels based on recent epidemiological surveys.

**Conclusions/Significance:**

The SEWI should be useful for estimation of schistosomiasis transmission surveillance, particularly with reference to the elimination of the disease in China.

## Introduction

**Figure 1:**
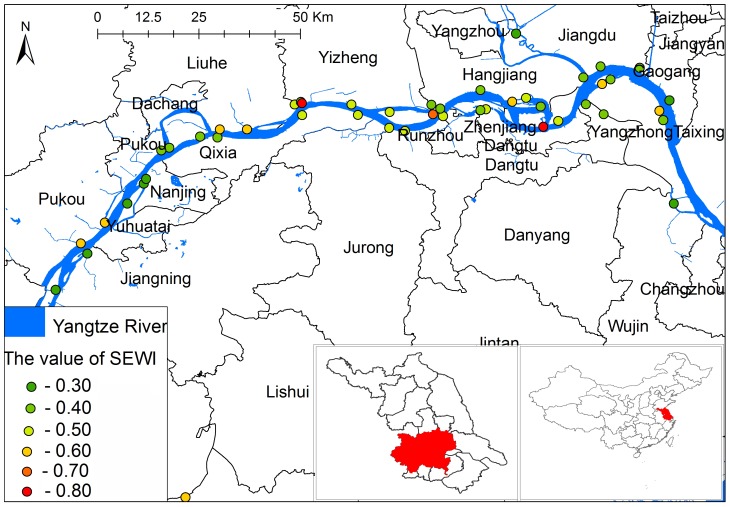
Location of the study area in Jiangsu Province, China, and the distribution of risk of schistosomiasis transmission base on SEWI.

Jiangsu Province is located in the lower reaches of the Yangtze River along the East Coast of China (Figure 1), where schistosomiasis is endemic. The disease is caused by the zoonotic trematode worm *Schistosoma japonicum* with the *Oncomelania hupensis* snail serving as the intermediate host for the parasite [1], [2]. Historically, Jiangsu Province was among the endemic areas with the highest level of schistosomiasis transmission in China. Due to extensive control efforts with a particular focus on intermediate host control in the past three decades, the province reached the criterion of transmission control in 1976, and no acute cases were reported between 1978 and 1984 [3], [4]. After that, various environmental changes (e.g. frequent flooding) altered the level of disease endemicity; snail habitats re-established or expanded resulting in re-emergence of highly endemic areas and reappearance of acute human cases [5], [6], [7]. In 2005, the government of Jiangsu Province revised and strengthened the schistosomiasis control strategies by the implementation of a package of comprehensive measures, such as integrated chemotherapy with praziquantel for humans and bovines, snail control, improved water supply, enhanced sanitation with focus on personal hygiene and health education [8]. After 5 years of extensive implementation of this comprehensive control program, all endemic counties in Jiangsu Province reached the criterion of transmission control (i.e. <1% human/bovine prevalence and absence of snail infection) [9]. After achieving this level of control again, the province embraced the next control objective – elimination of schistosomiasis transmission by 2020 [10]. Yet, challenges on route to this goal are present as traditional surveillance (e.g. focusing on infected humans and snails) is inadequate due to limited sensitivity of current diagnostic tools, which cannot support control strategies tailored to the new low-transmission situation [11]. In addition to host-based diagnostic methods, the use of mice challenge to assess environmental risk (the mouse bioassay), is widely used [12]. However, also the sensitivity of the mouse bioassay becomes unreliable when transmission levels decrease. These limitations present formidable challenges for surveillance and control, which have yet to be overcome, so a sensitive and field-friendly alternative is very much needed.

The Delphi method, originally developed by the Rand Corporation in 1948 [13], is a structured communication technique used for studying a system by soliciting opinions of a panel of experts, typically using two or more rounds of questionnaires. Because it is usually administered by mail, the likelihood of individual experts influencing each other is minimized at the same time as participation of experts from a large geographical area is enabled [14]. The Delphi method has been widely used for business forecasting and has some advantages over other forecasting methods [15], [16], [17], [18]. It has also been used successfully in other areas ranging from technology forecasting to drug abuse [13], [19], [20] as well as development of indicators d in early warning systems (EWS) for the spread of infectious diseases, such as the severe acute respiratory syndrome (SARS) epidemic and influenza outbreaks [21], [22], [23].

Although a single index (e.g. based on prevalence of human/bovine and/or snail infections) capturing the endemicity status of the disease would be attractive, schistosomiasis transmission is complicated and therefore challenging. We aimed at developing a comprehensive, field-friendly index capable of capturing the multi-faceted aspect of transmission that can be used to predict risk trends, particularly in low-transmission areas. Here we explore the use of the Delphi method to develop a Schistosomiasis Early Warning Index (SEWI) and assess its utility in Jiangsu Province of the lower Yangtze River.

## Materials and Methods

### Study area

The study area covered 19 endemic counties in the marshlands along the Yangtze River within Jiangsu Province. Fifty monitoring sites were selected to apply and evaluate the SEWI based on the potential transmission factors such as docking areas of mobile boatmen and fishermen in environments containing snails; sites where infected snails have been found in the past three years; and the administrative boundaries of the villages investigated (Figure 1). Permission for these activities was obtained from the local center for disease control and prevention.

### The study framework

**Figure 2:**
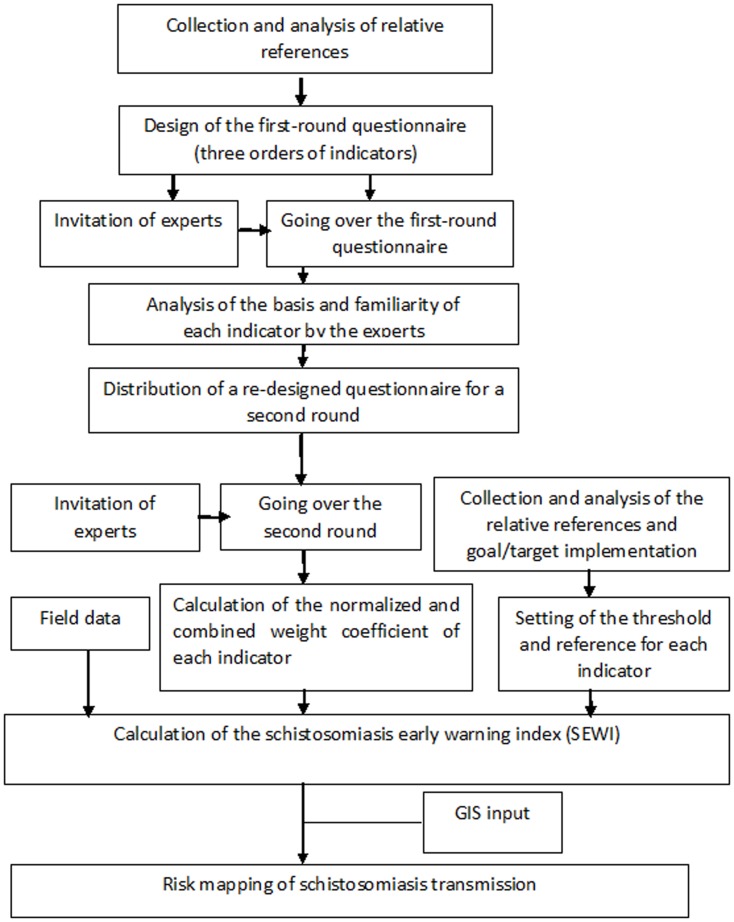
The framework of the whole study in Jiangsu Province, China.

Figure 2 shows the study framework. Firstly, relative references were collected and analyzed. Secondly, the questionnaire (containing three different orders of indicator on schistosomiasis transmission and control) was developed and experts invited to answer the questionnaire two times. Thirdly, the normalized and combined weights coefficient of each indicator was estimated, and the threshold of each indicator was set. Finally, the SEWI for each endemic county in Jiangsu Province was calculated and risk maps drawn up by support of geographical information systems (GIS) in order to visualize the different endemicity levels in a geographical format.

#### Development of indicators and questionnaire

Using the keywords “Schistosomiasis" and "Risk factors", we collected relevant articles from the Chinese literature database “China National Knowledge Infrastructure” (CNKI, http://www.cnki.net) since 2001, and also searched for relevant reports in archives, laws and protocols of schistosomiasis control (e.g. the National Standard of Schistosomiasis Control and Elimination (GBI5976-2006), the Work Specifications and Manuals of Schistosomiasis Prevention and Control (third edition) and the Comprehensive Management Plan for Key Projects of Schistosomiasis from 2009 to 2015). We then assessed the present situation of schistosomiasis transmission and control, monitoring and surveillance, including any possible use of any early warning indicators for schistosomiasis transmission proposing a basic framework of monitoring and early warning indicator system. Based on this literature review, we developed a set of questions for the questionnaire. Demographic (e.g. sex, age) and professional (e.g. education, current job title, employment history, and current employer) information regarding the experts was also collected.

In selecting indictors, two important characteristics were specifically considered – necessity/importance and maneuverability/feasibility. The indicator should reflect important characteristics of schistosomiasis transmission and be capable of signaling early transmission and information for each indicator should be collected or obtained with relative ease. The importance of each indicator was scored from 1 (least important) to 10 (very important).

Two factors were considered in assessing the authority of the experts to be interviewed: their knowledge foundation and their familiarity with judging each indicator. The former depends mainly on an the expert's theoretical foundation, practical experience and intuitive understanding of the field, in which he/she is expected to comment, while the latter is the degree each expert understands the measurement methods as well as contents and meaning of each indictor. The authority summary is the average of the knowledge foundation and the familiarity score, which can vary from very high (1) over medium (0.8) and low (0.6) to very low (less than 0.2).

#### The panel of experts

*Thirty* 30 schistosomiasis experts were chosen to participate in the study. The selection criteria for these experts included: (a) originated from schistosomiasis endemic areas (e.g. with different transmission levels and ecologies); (b) more than eight years' experience with work on schistosomiasis prevention, control and/or scientific research; (c) being a senior professional (a head of department of schistosomiasis control or research or with a high- to medium-level academic title); and (d) interested in this study and able to participate in all questionnaire surveys but without professional background as given above. About 80% of the experts chosen had been engaged in schistosomiasis control for more than ten years, 80% of them had senior titles and 73% were heads of county-level departments of schistosomiasis control. Their ages ranged between 28 and 54 with an average age of 42 years.

#### Implementation of the survey

Two rounds of interview were carried out. The experts were asked to consider whether the indicators were practical, feasible, and measurable for the schistosomiasis endemic areas. The first round was for the selection and optimization of indicators based on the following criteria (a) median score <7; (b) standard deviation >4; (c) coefficient of variation >50%; and (d) the addition of new indicator(s) based on the suggestions of more than half the number of the experts consulted. The second round was for establishing the SEWI after the first round revision.

### Statistical analysis

The expert responses were entered into a database and the necessity, feasibility and sensitivity of indicators as well as the authority degree of experts were then evaluated. The normalized weights and combination weights for all indicators were estimated as follows:

For each indicator (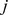), the authority coefficient (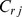), which expresses the degree of authority, was calculated as the arithmetic mean of the coefficients of judgment and familiarity:



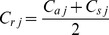
(Eq1)where *C_aj_* is the coefficient of the arithmetic mean of judgment for indicator *j* and *C*_s*j*_ the coefficient of the arithmetic mean of familiarity with regard to indicator *j* .

The normalized weight coefficient *W_j_* for indicator *j* was estimated as



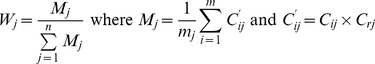
(Eq2)where *M_j_* is the weighted average of indicator *j*, *C'_ij_* the weighted score of expert *i* for indicator *j*, *C_ij_* the score of expert *i* for indicator *j*, while *n* is the number of indicators in the same order and *m_j_* the number of experts involved.

The combination weights 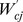 is



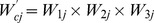
(Eq3)where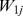, 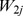 and 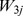 are the normalized weight coefficients of indicator *j* at the first, second and third order, respectively.

### Development and application of the SEWI

The Schistosomiasis Early Warning Index (SEWI) is an integrated index to evaluate the risk of schistosomiasis transmission and its value varies between 0 and 1, where the former indicates that the risk is zero and the latter that it is very high. The level was divided into four categories, namely very high risk at SEWI >0.8, high risk (SEWI >0.6), medium risk (0.3<SEWI<0.6) and low risk (SEWI<0.3). First, the threshold and standard reference was set for each indicator, and the real value of each indicator was normalized to only move between 0 and 1. If the indicator is qualitative, such as “Yes” or “No”, the value was set as “1” or “0”. Some indicators express a proportion of the ideal, in which case it should be divided by the value of an implementation goal or target, while other values such as the infection rate of humans or that of livestock can be directly used.

Second, the true value of each indicator was collected from the field for the study period between 2010 and 2012. Third, the SEWI value of each point was calculated using the following formula



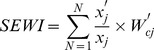
(Eq4)where 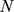 is the number of indicator, *x'_j_* is the true value of the indicator *j*, 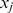 is the threshold and standard reference value of the indicator *j*, and 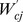 is the combination weight of the indicator *j*. The formula for the protective factors *f*, such as *Control measures*, was different from others, namely,



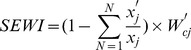
(Eq5)Fourth, we redefined the risk of schistosomiasis transmission based on the SEWI value and mapped the distribution of the regions at risk. Table 1 summarizes the calculation steps of SEWI. Finally, the risk map was compared with the current endemicity levels (e.g. endemic, transmission control, and transmission interruption) based on field epidemiological surveys to qualitatively assess performance of the new index.

**Table 1:** The characteristics and standardized or reference value of each indicator and the demonstration calculation process of SEWI in Jiangsu province, China.

First-order indicator (Weight)	Second-order indicator (Weight)	Third-order indicator	The standardized or reference value	Weight a	Value of example point b	SEWI calculation c
A: Endemic status (0.366)	A1: Endemic status of human (0.129)	A11: The local infectious case of schistosomiasis within last three years	Whether there exists the local case? Yes = 1; and No = 0	0.0431	1	0.0431×1
		A12: The infection rate of schistosomiasis	The true infection rate	0.0424	0.1	0.0424×0.1
		A13: The acute case of schistosomiasis within last three years	Whether there exists the acute case? Yes = 1; and No = 0	0.0433	1	0.0433×1
	A2: Endemic status of snails (0.127)	A21: The area of snail habitat	The true value divided by the reference value of 70 thousand square meter	0.0143	40,000	0.0143×(40,000÷70,000)
		A22: The density of live snail	The true value divided by the reference value of 0.5 snail per 0.1 square meter	0.0144	0.2	0.0144×(0.2÷0.5)
		A23: The number of snail habitat	For the point evaluation: Whether there exist the distribution of snail? Yes = 1; and No = 0For the regional evaluation: The true value divided by the reference value of 5 which is the average number	0.0131	1	0.0131×1
		A24: The trend of snail area/density within last three years	Whether area/density increases? Yes = 1; and No = 0	0.0131	0	0.0131×0
		A25: The area of infected snail habitat	The true value divided by the reference value of 10 thousand square meter	0.0147	2 000	0.0147×(2 000÷10 000)
		A26: The density of infected snail	The true value divided by the reference value of 0.05 snail per 0.1 square meter	0.0151	0.2	0.0151×(0.2÷0.05)
		A27: The number of infected snail habitat	For the point evaluation: Whether there exists the distribution of an infected snail? Yes = 1; and No = 0. For the regional evaluation: The true value divided by the reference value of 2	0.0147	1	0.0147×1
		A28: The detection of an infected snail within last three years	Whether there exists the infected snail? Yes = 1; and No = 0	0.0145	1	0.0145×1
		A29: The area of emergence habitat of snail	For the point evaluation: Whether there exists the emergence habitat of snail? Yes = 1; and No = 0. For the regional evaluation: The true value divided by the reference value of 0.1	0.0136	1	0.0136×1
	A3: Transmission route (0.110)	A31: The result of sentinel mice survey	Whether there exist the infected sentinel mice? Yes = 1; and No = 0	0.0546	1	0.0546×1
		A32: The result of snail detection	Whether there exist the infected snails?	0.0555	1	0.0555×1
B: Control measures (0.343)	B1: Control measures for human (0.168)	B11:The cover rate of screening and chemotherapy of schistsosomiasis	The true value of cover rate (%)	0.0329	0.1	0.0329×(1–0.1)
		B12: The cover rate of monitoring high risk group	The true value of cover rate (%)	0.0348	0.7	0.0348×(1–0.7)
		B13: The storage of prevention material and drug	Whether there exists the storage? Yes = 1; and No = 0	0.0321	0.5	0.0321×0.5
		B14: The registration rate of the population contacting with the risk water	The true value of cover rate (%)	0.0306	0.2	0.0306×(1–0.2)
		B15: The immediacy of the treatment of the acute case	Whether it is immediate? Yes = 0; and No = 1	0.0375	0	0.0375×0
	B2: Control measures for snail (0.175)	B21: The completion rate of snail detection	The true value of cover rate (%)	0.0435	0.1	0.0435×(1–0.1)
		B22:The cover rate of snail killing	The true value of cover rate (%)	0.0451	0.3	0.0451×(1–0.3)
		B23: The effectives of snail killing	The true value of cover rate (%)	0.0444	0.9	0.0444×(1–0.9)
		B24: The cover rate of environmental change	The true value of cover rate (%)	0.0419	0.2	0.0419×(1–0.2)
C: Social and environmental factors (0.291)	C1: Geographical factors (0.147)	C11: The assembly centres of mobile boatmen and fishermen	Whether there exist? Yes = 1; and No = 0	0.0477	0	0.0477×0
		C12: The assembly centres of large enterprises, construction projects	Whether there exist? Yes = 1; and No = 0	0.0496	0	0.0496×0
		C13: The livestock grazing areas and waterways in direct connection with the Yangtze River.	Whether there exist? Yes = 1; and No = 0	0.0498	0	0.0498×0
	C2: Social factors (0.144)	C21: The popularizing rate of sanitary toilets	The true vale (%)	0.0275	0.8	0.0275×(1–0.8)
		C22: The cover rate of feces harmless treatment	The true vale (%)	0.028	0.9	0.028×(1–0.9)
		C23: The cover rate of individual protection	The true vale (%)	0.0263	0.98	0.0263×(1–0.98)
		C24: The local expenditure per capita for schistosomiasis control	The true value divided by the reference value of 1	0.0311	0.5	0.00311×(0.5÷1.0)
		C25: The guarantee of organization management and policy	Whether there exist? Yes = 0; and No = 1	0.0309	0	0.0309×0

a the combination weights of the third-order indicator;

b it is the just example, and the filed value come example point;

c the procedure showing the calculation of SEIW.

### Ethics statement

All studies described here were approved by the Ethics Review Committee of Jiangsu Institute of Parasitic Diseases, China (Permission number: JIPDERC2007008). The field studies did not involve endangered or protected species.

## Results

### Development of the indicators

In this study, more than 100 relevant peer-reviewed articles and reports from archives were collected, and key impact factors of schistosomiasis transmission were summarized. Reviewing these articles and reports indicate that the transmission pattern and impact factors are similar in the endemic areas along the Yangtze River, at least within Jiangsu Province. We pre-selected three first-order indicators, 11 second-order indicators, and 58 third–order indicators according to national control guideline, references and control experiences. After the first Delphi round, three first-order, seven second-order, and 30 third-order indicators were accepted as preliminary indicators and after the second round, the final indicators and the combination weights of each indicator were chosen (Table 1). The first-order indicators include three aspects relevant for transmission of schistosomiasis: *endemic status*, *control measures*, and *social and environmental factors* with *endemic status* as the most important one (i.e. having the highest values). For the seven second-order indicators, Snail c*ontrol measures* had the highest weight (0.175) and *Transmission route* the lowest (0.110).

### Reliability of the survey

#### Authority coefficient

Out of 30 effective questionnaires collected the response rate was 100% for the first round and 93% for the second round. The score ranges for judgment (*C_aj_*) and for familiarity (*C_sj_*) were 0.835–0.935 and 0.756–0.939, respectively, and the corresponding arithmetic means 0.895 and 0.886, while the authority coefficient (*C_rj_*) range was 0.800–0.928 and mean was 0.891. Table 2 shows the authority coefficient of the first-order indicators. The survey authority of the expert consultation was higher, indicating that the prediction precision of the survey should also be higher.

**Table 2:** The authority and necessity of the first-order indicator in Jiangsu Province, China.

First-order	The authority of the indicators a			The importance of indicators b		
indicator	Judgment coefficient( 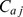 )	Familiar coefficient( 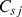 )	Authority coefficient( 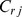 )	Weighted means of indicator	Standard deviation	Coefficient of variation (%)
Endemic status	0.91	0.89	0.90	9.00	0.82	9.94
Control measures	0.89	0.85	0.87	7.65	0.94	12.17
Social and environmental factors	0.79	0.88	0.83	8.55	1.17	17.76

a The summary authority is the average of the judgment coefficient and the familiarity;

b The indicator reflects important characteristics of schistosomiasis transmission.

#### The consistency coefficient

Table 2 also shows that the importance of the first-order indicators after the second round with the standard deviation and coefficient of variation of the *Social and environmental factor* being greater than those of the other indicators. However, the standard deviation and coefficient of variation still remained relatively low. The consistency coefficient increased from 0.54 to 0.84 indicating that the opinion of each expert became consistent after the second round [14].

#### Risk prediction based on the SEWI

Figure 1 shows the redefined distribution the risk of schistosomiasis along the Yangtze River based on SEWI values; the risk in the most parts of study area was medium with high snail density, particularly near islands in the Yangtze River. High and low risk areas were also verified by the endemicity classifications, namely transmission control and transmission interruption, based on field epidemiological surveys.

## Discussion

Transmission of *S. japonicum* is complicated, involving a variety of biological and social factors. The social factors include those at the national and regional level, such as policies and patterns of development, which impact local economic activities that in turn influence risk factors of human infection at the community level including households and individuals [24], [25], [26]. Huang et al. [6] reviewed how social structure and factors influence individual risk and prevalence of *S. japonicum* infection in China, and showed that the risk of *S. japonicum* infection is also influenced by the domestic environment, including both location of the house in relation to snail-colonized water sources, access to safe water and improved sanitation. The risk of *S. japonicum* infection is thus related to many different factors, not only environmental but also social ones [27], [28], [29], [30]. Early warning identification and control measures should be considered as multi-faceted. However, a limited resource allocated to the schistosomiasis control program in Jiangsu Province is basically a local activity, unable to implement different interventions simultaneously [9], [10], [31]. This fact has drawn attention by both policy-makers and medical personnel, who advocate a multi-faceted approach for schistosomiasis elimination [32], [33], [34], [35]. Thus, it is necessary to establish priorities and integrated assessment methods based on both environmental and social factors. We first proposed the SEWI concept to identify relevant types of data needed in estimating the impact of schistosomiasis interventions on the transmission risk; we also tested the operational feasibility of SEWI to develop a conceptual framework and identify obstacles that need to be overcome. Because transmission patterns and impact factors are different in different environmental regions, we considered various local regions along the Yangtze River when developing the methodology. However, we believe that the methodology as proposed can also be applied to other endemic areas of schistosomiasis.

The Delphi method has been used as a tool to implement multi-stakeholder approaches in developing countries. Recently, an increasing number of studies on the application of the Delphi method to schistosomiasis have been reported. Yu et al. [36] established three first-order indicators, namely the infection rate of humans, livestock and snails, and six second-order indicators, to determine the weight coefficient in the control effect evaluation, while Luo et al. [37] and Yu et al. [38] established the effect-evaluation index system of schistosomiasis in the mountainous region of Yunnan Province and the marshland region of Hubei Province, respectively. Xu et al. [39] established an objective, operational assessment system or the provision of scientific evidence for revising the current, official ‘Criteria for Control and Elimination of Schistosomiasis in China’ (GB 15976-2006). However, the aims of these previous studies were different from ours and the scope was limited to specific, research purposes. Rather than predict the risk of transmission in a prospective manner, they were generally carried out to evaluate the effectiveness of control measures and did not aspire to establish an integrated index by collecting and combining field data. In addition, the endemic status of schistosomiasis along Yangtze River in Jiangsu Province is characterized by low prevalence rates and generally low/intensity infections, which is significantly different from other regions. Here we aim to develop a comprehensive and feasible index, the SEWI, and demonstrate its use in Jiangsu Province, representing a low-transmission area along the lower Yangtze River.

The first-order indictors remained the same after the second round, i.e. *Endemic status*, *Control measures*, *Social and environmental factors* and the weight value of each indicator were also similar. The coefficient of variation of *Social and environmental factors* was larger than the other two indicators, indicating that the risk contribution of these factors with respect to *S. japonicum* infection was different [6], [40], [41]. The number of second-order indicator decreased from 11 to 7; four indicators were removed after the second round,two indicators were close to the livestock, including the *Endemic status of livestock*, *Control measures for livestock*. The other two indicators were *Health resources* and *Climate factors*. Throughout history, livestock (particularly water buffaloes) have been the main source of infection for schistosomiasis [42], [43]. The economy of Jiangsu Province has been developing rapidly since the 1990s, and the province is now one of the most economically advanced in the country. During this time period, the cattle population decreased significantly and pasture in the snail habitats was forbidden. The infection rate of livestock also declined significantly year-by-year and no positive stool tests have been observed in livestock since 2008. Thus, livestock as source of infection has weakened significantly in Jiangsu Province [10], [44], [45]. Given the situation, during the 1^st^ round expert consultation, most experts suggested removing the indicators related to livestock. The economic and meteorological characteristics in the 18 selected endemic counties were all similar, and most funds for control activities came from national and provincial agencies [46]. The contribution of health resource and meteorological factors to *S. japonicum* infection were similar, and most experts also suggested removing or changing these two second-order indictors. After two rounds, the weights of *control measures for snail* and *transmission route* were the highest and the lowest, respectively, suggesting that the contribution of the snail distribution was important for schistosomiasis transmission, which is consistent with the current control strategy in Jiangsu Province [10]. Among the third-order indicators, the weight value of the *result of snail detection* was the highest and showed that each county along the Yangtze River should strengthen the snail surveillance component of their control efforts to lower the risk of schistosomiasis transmission.

From 2005, the government of Jiangsu Province revised the schistosomiasis control strategy to emphasize integrated measures aimed at reducing the transmission of *S. japonicum*. The human seroprevalence declined by 64% and the parasitological prevalence by 58% from 2005 to2008 [10]. However, there remains a potential for re-emergence of the disease in the study area as infected snails and human infections continue to be reported in the region [7], [47]. Developing a EWS to inform about the risk for schistosomiasis transmission, particularly in low-transmission areas, has become highly desirable. World Health Organization (WHO) is strengthening existing surveillance systems for infectious diseases developing EWSs based on new concepts and techniques in order to trigger prompt public health interventions. We have explored an EWS based on mice bioassays and spatio-temporal analysis [12] constructing a comprehensive index that integrates all possible risk factors related to *S. japonicum* transmission. Furthermore, the method can be adapted and applied also for other diseases following the study framework shown in Figure 2. However, more technical development is still needed. Firstly, the number of indicators can be optimized or simplified without losing informative power thereby increasing the specificity of the SEWI; Secondly, it would be useful to carry out a prospective study to further evaluate and verify the SEWI approach.

## Conclusion

An EWS framework has been has been developed through two rounds of a Delphi expert consultation process based on literature search and documentation. A sensitive index (SEWI) capable of schistosomiasis monitoring and surveillance is described and successfully applied for mapping of the distribution of the risk of *S. japonicum* infection at a time when monitoring and surveillance are needed for the elimination of the disease.
